# Adsorption of hydroxamate siderophores and EDTA on goethite in the presence of the surfactant sodium dodecyl sulfate

**DOI:** 10.1186/1467-4866-10-5

**Published:** 2009-06-13

**Authors:** Naraya Carrasco, Ruben Kretzschmar, Jide Xu, Stephan M Kraemer

**Affiliations:** 1 Institute of Biogeochemistry and Pollutant Dynamics, Department of Environmental Sciences, ETH Zurich, CHN, CH-8092 Zurich, Switzerland; 2 Chemistry Department, University of California, Berkeley, California, 94720-1460, USA; 3 Department of Environmental Geosciences, University of Vienna, Althanstrasse 14, A-1090 Vienna, Austria

## Abstract

Siderophore-promoted iron acquisition by microorganisms usually occurs in the presence of other organic molecules, including biosurfactants. We have investigated the influence of the anionic surfactant sodium dodecyl sulfate (SDS) on the adsorption of the siderophores DFOB (cationic) and DFOD (neutral) and the ligand EDTA (anionic) onto goethite (α-FeOOH) at pH 6. We also studied the adsorption of the corresponding 1:1 Fe(III)-ligand complexes, which are products of the dissolution process. Adsorption of the two free siderophores increased in a similar fashion with increasing SDS concentration, despite their difference in molecule charge. In contrast, SDS had little effect on the adsorption of EDTA. Adsorption of the Fe-DFOB and Fe-DFOD complexes also increased with increasing SDS concentrations, while adsorption of Fe-EDTA decreased. Our results suggest that hydrophobic interactions between adsorbed surfactants and siderophores are more important than electrostatic interactions. However, for strongly hydrophilic molecules, such as EDTA and its iron complex, the influence of SDS on their adsorption seems to depend on their tendency to form inner-sphere or outer-sphere surface complexes. Our results demonstrate that surfactants have a strong influence on the adsorption of siderophores to Fe oxides, which has important implications for siderophore-promoted dissolution of iron oxides and biological iron acquisition.

## Introduction

**Figure 1:**
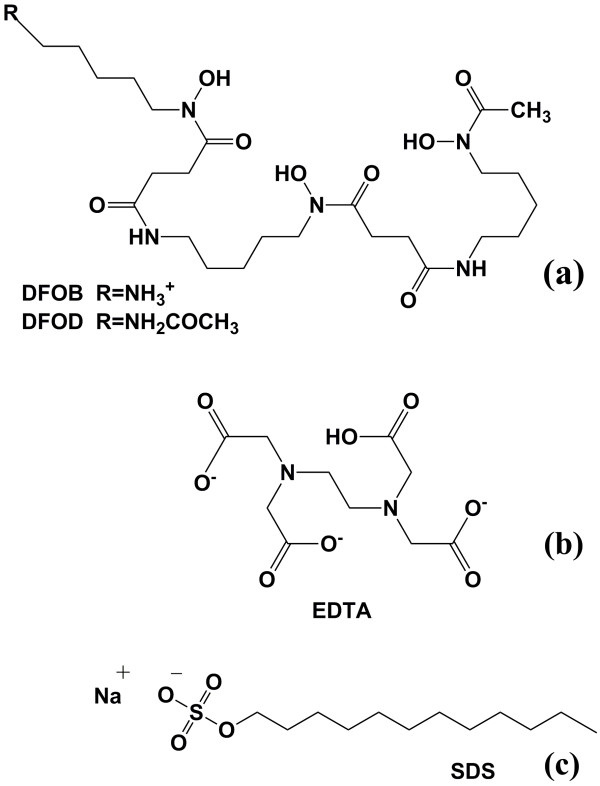
**(a) Structure of the siderophores desferrioxamine-B (DFOB) and desferrioxamine-D (DFOD).** The terminating group R of DFOB is an amine group with a pK_a_ of 8.4. The three hydroxyl groups have pK_a_ values of 8.73, 8.99, and 10.1, respectively [11]. The terminating group R of DFOD is acetylated and has no charge. The stability constant for Fe-DFOB and Fe-DFOD are 10^30.7^ and 10^30.76^, respectively (I=0.1M) [7]. (b) Structure of EDTA with pK_a_ values of 2, 2.69, 6.13, and 9.52 [31]. The dominant species under the experimental conditions is doubly negatively charged. (c) Structure of the sodium salt of the anionic surfactant dodecyl sulfate (SDS) with a pK_a_ of 2.3 [22].

The bioavailability of Fe(III) in oxic soils, sediments, and surface waters at near-neutral pH is limited by the low solubility and slow dissolution rates of iron oxides and hydroxides. In order to overcome this low iron solubility, many microorganisms and roots of graminaceous plants exude highly Fe(III)-specific, low-molecular weight (0.5 to 1.5 kDa) ligands, the a group of compounds called siderophores [1-5]. Hundreds of structurally distinct siderophores are known, typically with ligating catecholate, carboxylate, α-hydroxycarboxylate, or hydroxamate functional groups [2,6]. Most siderophores are hexadentate and form 1:1 Fe(III)-complexes [7-10]. Desferrioxamine-B (DFOB), presented in Figure 1, is an example of a cationic (pH<8) trihydroxamate siderophore found in both terrestrial and marine systems [11]. Sub-micromolar siderophore concentrations have been observed in soil solutions [12]. The role of siderophores in enhancing the bioavailability of iron depends on its specificity for iron. For example, even in soils in equilibrium with calcium carbonate, where otherwise strong competition between Ca and iron for complexation would be expected, the dominant soluble species of the iron specific DFOB is the free, fully protonated ligand.

An important function of siderophores in biological iron acquisition is the acceleration of iron oxide dissolution [13]. Organic ligands and their complexes can adsorb onto iron oxides by forming inner-sphere and/or outer-sphere surface complexes. For simplicity, the term "outer-sphere" will be used here to include surface species which are sorbed by H-bonding with protonated surface hydroxyl groups or by hydrophobic interactions with adsorbed surfactant molecules. The adsorption of the ligand by formation of inner-sphere complexes between the ligand and structural iron at the oxide surface is usually considered to be the first step of a ligand-promoted dissolution mechanism [14]. This conclusion has been drawn based on observations of a linear correlation between the rate of ligand-promoted dissolution and the amount of adsorbed ligand, whereas the relationship between soluble ligand concentrations and dissolution rates has the same shape as the corresponding adsorption isotherm. This led to the formulation of a simple rate law:(1)

where the surface area normalized dissolution rate R [mol s^-1 ^m^-2^] depends linearly on the adsorbed ligand concentration L_ads _[mol m^-2^] with a rate constant k [s^-1^]. The conceptual model underlying this rate law is based on the assumption that the formation of coordinative bonds between the inner-sphere adsorbing ligand and a surface metal center will kinetically labilize other bonds in the coordination sphere of the metal center and thereby promote the bond exchange reactions involved in its dissolution [14]. Indeed, the effect of inner sphere coordinating ligands on dissolution rates of simple oxides is well correlated to their effect on water exchange rates around corresponding metal centers in solution [15]. However, it is important to keep in mind that several surface sites and corresponding inner-sphere surface complexes with contrasting kinetic properties may exist [14]. Therefore, Equation 1 is only valid as long as this surface speciation is constant.

Studies on siderophore-promoted dissolution of iron oxides [13,16,17] and direct spectroscopic evidence [18] have suggested that siderophores can form inner-sphere surface complexes on iron oxide surfaces, in addition to possible outer-sphere complexes.

Surface-active agents (surfactants) are ubiquitous in natural systems [19,20] and may potentially influence the free energy change of adsorption of siderophores [21]. Surfactants are structurally diverse organic compounds with amphiphilic properties arising from a hydrophobic hydrocarbon chain (tail) and hydrophilic (head) structural moieties in the same molecule [22]. The head groups can be anionic, cationic, non-ionic, or zwitterionic (i.e., bearing both positive and negative charge). Synthetic surfactants occur in the environment as contaminants due to their widespread use in industrial processes and as household detergents. In soils, bio-surfactants are produced by a range of microorganisms [19,20] and their production can be triggered by nutrient limitations [23,24]. Root mucilage was also found to contain powerful bio-surfactants that affect biogeochemical and physical processes in the rhizosphere [25].

Adsorption of ionic surfactants on oppositely charged mineral surfaces has been the subject of intense research [26-30]. It is well accepted that anionic surfactant monomers adsorb on oppositely charged surfaces by electrostatic and specific forces at low surface coverage. With increasing surfactant concentrations, surfactant molecules form hemimicelles (monolayered clusters) or admicelles (bilayered clusters) on the surface by a combination of electrostatic and hydrophobic forces. With further increasing concentrations, adsorption eventually reaches a maximum at which mainly admicelles are present. This adsorption maximum is often reached near the CMC, i.e., the surfactant concentration above which micelle formation occurs in solution. Surfactant adsorption on mineral surfaces changes the physico-chemical properties of the interface. For instance, at low concentrations, hemimicelles with surfactant heads close to the surface and tails directed toward the solution increase the hydrophobicity of the surface. Admicelles with a second layer of adsorbed surfactant with the head groups pointed toward the solution lead to surface charge reversal. As an example, the adsorption isotherm of the anionic surfactant sodium dodecyl sulfate (SDS) onto goethite has been presented in a previous paper [21].

The dramatic effects of adsorbed surfactants on surface charge and hydrophobicity may have important implications for the adsorption of siderophores and their iron complexes. In a previous study [21], we observed that the adsorption of DFOB to goethite increased with increasing concentrations of sodium dodecyl sulfate. Moreover, the presence of low surfactant concentrations in the low micromolar range caused a 3-fold acceleration of the dissolution rates of goethite.

The overall standard free energy change of adsorption of ions or molecules can be conceptually divided into various free energy contributions including free energy of chemisorption, electrostatic interactions, hydration and dipole-orientation, and hydrophobic interactions. In order to better understand the nature of the interactions responsible for siderophore adsorption in the presence of surfactants, we studied the influence of SDS on the adsorption of three complexing ligands and their respective iron complexes to goethite. We chose ligands with contrasting charge and hydrophobicity in order to explore how electrostatic and hydrophobic interactions with surfactants affect their adsorption. The dominant H_2_EDTA^2- ^species at pH 6 is highly hydrophilic and negatively charged. The two siderophores contain a hydrophobic pentyl chain that is (at the experimental pH) terminated by a positively charged amine group in the case of DFOB^+^. This group is acetylated and uncharged in the case of DFOD^0^, a synthetic derivate of DFOB (Figure 1a). In the following discussion, the charge of the main species will be indicated wherever relevant. Considering the structural distance of the modified terminal group to the hydroxamate groups of DFOB and DFOD, we assume that this modification has little effect on the properties of the ligating groups. This assumption is supported by the similarity of stability constants for their iron complexes in solution (see caption Figure 1) [7]. Therefore, the chemical interactions with surface sites (chemisorption) were expected to be similar for both siderophores. Consequently, we assume that any major differences in the effect of co-adsorbed surfactants on DFOB and DFOD adsorption can be assigned to free energy contributions caused by electrostatic or hydrophobic interactions. The third ligand, EDTA (ethylenediaminetetra-acetic acid, Figure 1b), was chosen because of its negative charge and strong hydrophilicity [31]. All three ligands accelerate iron oxide dissolution by ligand-promoted dissolution mechanisms. Therefore, we also studied the adsorption of 1:1 iron-ligand complexes that are products of the dissolution process. SDS was chosen as a model for anionic surfactants occurring in soils. It should be noted that SDS does not capture the structural diversity of biosurfactants. However, SDS has been widely used in studies of the behavior of surfactants on mineral surfaces and provides a valuable basis for the understanding of their effect on ligand adsorption.

## Experimental methods

### Preparation of Goethite

Goethite (α-FeOOH) was synthesized following Böhm's method as described in Schwertmann and Cornell [32]. Briefly, 100 mL of freshly prepared 1 M Fe(NO_3_)_3_·9H_2_O were mixed rapidly with 180 mL of 5 M KOH in a polypropylene beaker. All solutions used in this study were prepared with high-purity water (Milli-Q, Millipore, 18 MΩcm). The precipitated hydrous ferric iron was immediately diluted to 2 L with deionized water and heated in closed polypropylene flasks at 70°C for 60 hours. Afterwards, the precipitate was resuspended in high-purity water, centrifuged, and decanted. This washing procedure was repeated several times until the supernatant was free of chloride and then freeze-dried. X-ray powder diffraction confirmed the formation of α-FeOOH. The specific surface area was determined as 38 m^2^g^-1 ^by a multipoint N_2_-BET adsorption method (surface area analyzer Gemini 2360, Micromeritics, Belgium).

### Synthesis of desferrioxamine-D (DFOD)

Prior to the synthesis of DFOD, N-acetyl-O,O,O-triacetyl-desferrioxamin was synthesized as follows. DFOB (6.4 g, 10 mmol) and ground anhydrous K_2_CO_3 _(3 g, 20 mmol) were mixed with H_2_O-free N,N-dimethylformamide (25 mL). This suspension was heated to 70°C under nitrogen atmosphere for 30 min, then cooled to room temperature and diluted with CH_2_Cl_2 _(100 mL). To this suspension, a solution of acetic anhydride (4.1 g, 40 mmol) in 20 mL CH_2_Cl_2 _was slowly added over a period of 30 min. The reaction mixture was stirred for 4 h and filtered. The filtrate was successively extracted with water (2 × 50 mL), saturated aqueous NaHCO_3 _solution (50 mL) and aqueous sodium chloride solution (50 mL) and then dried over anhydrous Na_2_SO_4_. The solvents were removed *in vacuo*, and the N-acetyl-O,O,O-triacetyl-desferrioxamine was obtained as colorless thick oil (yield 6.7 g, 9.2 mmol, which correspond to 92% of the theoretical obtainable amount). Then, the N-acetyl-O,O,O-triacetyl-desferrioxamine was dissolved in 100 mL of methanol, cooled to 0°C with an ice bath, and the solution was saturated with gaseous ammonia. The solution was kept at room temperature for 4 h, and then refrigerated overnight. Crude DFOD was collected by filtration and re-crystallized twice from methanol/water for purification. Pure DFOD was obtained as colorless crystalline material. 4.8 g of DFOD were obtained corresponding to 84.7% of the theoretical obtainable amount. The melting point was 180 ± 1°C. The relative composition of elements for C_27_H_50_N_6_O_9 _was found to be: C, 53.93 (53.84); H, 8.44 (8.32); N, 13.76 (13.95)% (theoretical values are given in parenthesis). Detailed information on the characterization of DFOD is provided in additional file 1.

### Adsorption of free ligands in the presence of SDS

The adsorption of EDTA (Fluka, Switzerland) and the siderophore DFOD to goethite as a function of increasing SDS (Fluka, Switzerland) concentrations was studied at pH 6 in 40 mL batch reactors. SDS concentrations were kept well below the critical micelle concentration (CMC = 3.1 mM at 25°C in 0.01 M NaCl; [33]). 30 mL batches of goethite (α-FeOOH) suspensions (solids concentrations of 7 g/L; pH 6.0 ± 0.05; 0.01 M NaClO_4 _(as ionic strength buffer)) containing SDS at various concentrations were prepared and pre-equilibrated for 68 hours on an end-over-end shaker at 25 ± 1°C. Then, 10 mL of stock solutions containing 320 μM EDTA, or DFOD in 0.01 M NaClO_4 _adjusted to pH 6.0 ± 0.05 were added to the suspensions. The total concentrations of EDTA, or DFOD were 80 μM in all batches and the total SDS concentrations varied from 0 to 600 μM. The suspension pH was measured using a pH electrode (Metrohm, 6.0234.100) and readjusted, if necessary, to pH 6.0 ± 0.05 by small additions of NaOH or HCl. All reactors were wrapped in aluminum foil to avoid photochemical reactions. After 24 hours equilibration time on an end-over-end shaker at 25 ± 1°C, 5 mL aliquots of each suspension were filtered through 0.025 μm cellulose nitrate membranes using polycarbonate syringe filter holders (25 mm diameter, Schleicher & Schuell, Germany). The concentration of adsorbed ligands was determined indirectly by measuring the ligand concentration left in solution after filtration. DFOD was immediately analyzed after filtration with a UV-visible spectrophotometer (Cary 50 BIO, Varian, Australia) as an iron complex in the presence of an excess of Fe(III) at 432 nm as described in [34]. EDTA was also analyzed spectrophotometrically as iron complex following a procedure described by Bhattach et al. [35]. Blank experiments without goethite were performed to monitor any losses due to adsorption of the ligands to the vessel or filter, but no significant losses were observed. During the 24 h equilibration time after the siderophore addition, some ligand-promoted dissolution of goethite may have occurred. However, based on the low dissolution rates of goethite at pH 6 in the presence of EDTA [36] and siderophores [37], the expected dissolved iron concentrations are in the low micromolar range and are not expected to affect the results.

### Adsorption of Fe complexes in the presence of SDS

Stock solutions of 1:1 Fe-EDTA, Fe-DFOB and Fe-DFOD complexes (120 μM in 0.01 M NaClO_4_) were prepared by dissolving Fe(NO_3_)_3_·9H_2_O (Fluka, Switzerland) in a 0.01 M NaClO_4 _solution and adding EDTA, DFOB, or DFOD solutions at equimolar concentrations. The stock solutions were added to goethite suspensions pre-equilibrated with SDS at pH 6.0 ± 0.05 at 25 ± 1°C. The suspension pH was measured and readjusted if necessary to 6.0 ± 0.05 by small additions of NaOH or HCl. The experimental set up was similar to the adsorption experiments with free ligands as described above, except that the solids concentration was 2.5 g/L. The total concentration of Fe complexes in all reactors was 30 μM and the total SDS concentration was 0–600 μM. The equilibration time after the addition of the Fe complexes was 24 h. After filtration, the dissolved concentrations of iron complexes left in solution were immediately analyzed by inductively coupled plasma optical emission spectrometry (ICP-OES, Vista-MPX, CCD Simultaneous, Varian, Australia) at the wavelength 238.2 nm. In some experiments, the concentrations of 1:1 Fe complexes were also analyzed by UV-visible spectrophotometry. The lack of difference between these two methods indicated the formation of 1:1 Fe-ligand complexes (see additional file 2). In addition, Fe-DFOB adsorption isotherms were recorded using a similar set-up in the presence of 0, 15, and 800 μM total SDS, respectively. Samples were taken 24 hours after the addition of the iron complex, filtered, and iron concentrations were analyzed by ICP-OES.

## Results and discussion

### Adsorption of free ligands

**Figure 2:**
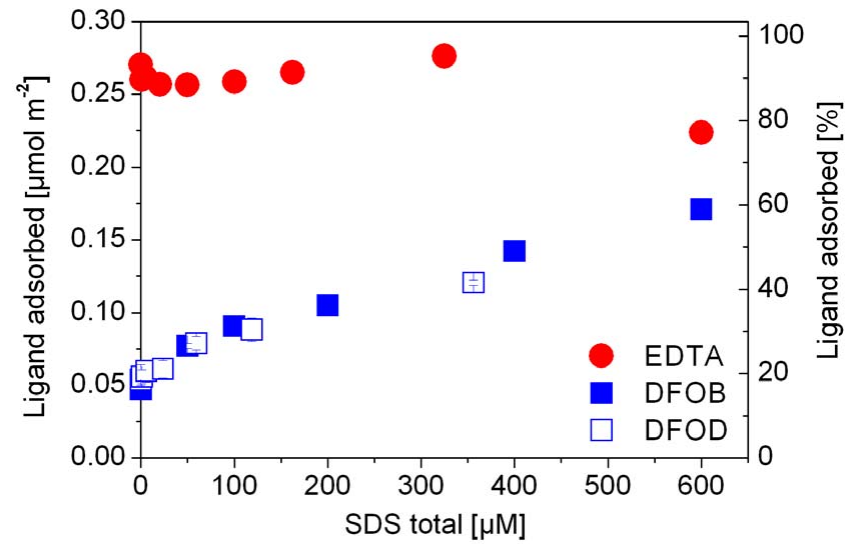
**Adsorption of EDTA and the siderophores DFOB (data from **[21]**) and DFOD onto goethite at pH 6 as a function of SDS concentration (0.01 M NaClO_4_, 7 g/L goethite, 24 h equilibration time).** The total ligand concentration was 80 µM. Error bars indicate the standard deviation of three replicates.

The influence of increasing SDS concentrations on the adsorption of EDTA and the siderophores DFOB (data from [21]) and DFOD is shown in Figure 2. SDS had almost no effect on the adsorption of EDTA, except for a slight decrease of EDTA adsorption at the highest SDS concentration (600 μM). Considering the hydrophilic nature of EDTA, hydrophobic interactions with co-adsorbed SDS are not expected. The doubly deprotonated H_2_EDTA^2- ^(pK_a3 _= 6.13) is the dominant species in solution in the absence of soluble iron at pH 6. Based on sorption and titration experiments combined with surface complexation modeling, Nowack and Sigg [38] proposed that the dominant surface species of EDTA at pH 6 is a triply deprotonated, singly charged binuclear inner-sphere surface complex (≡ Fe_2_EDTA^-^)

ATR-FTIR studies have conclusively shown that sulfate forms inner-sphere and outer-sphere surface complexes on goethite, depending on solution pH [39] At pH 6 and above, the dominant surface species was an outer-sphere surface complex, while at low pH sulfate was sorbed mainly as inner-sphere complex. Therefore, it seems likely that the sulfate head groups of SDS will also mainly form outer-sphere complexes at pH 6. Hence, little adsorption competition between SDS and EDTA is expected at low surfactant concentrations. Indeed, SDS had little effect on EDTA sorption at concentrations < 600 μM total SDS (Figure 2). Nevertheless, it is possible that adsorbed SDS forms a minor inner-sphere surface species via the sulfate head groups that may compete with EDTA for surface sites at the highest SDS concentration leading to the observed decrease of EDTA adsorption (Figure 2).

One could also assume that SDS may influence EDTA adsorption by modifying the charge at the mineral-water interface. Electrophoretic mobility measurements showed a reversal from positive to negative values upon formation of a surfactant bilayer on goethite [21]. However, electrophoretic mobility measurements probe the electrical potential at the plane of shear, which is beyond the surfactant (bi)layer, i.e., at greater distance from the mineral surface. Under the assumption that EDTA forms only inner-sphere complexes and that the charge of adsorbed EDTA is located at or close to the surface its adsorption does not depend on variations of the potential above the hemimicelles and admicelles.

The surface potential depends, among other factors, on the protonation state of the surface and it has been observed that the adsorption of surfactants, especially at low concentrations, increases the protonation of oxide surfaces [40,41]. However, Nowack and Sigg [38] have reported that the adsorption of EDTA as a binuclear complex on goethite is constant between pH 5 and 7, which is consistent with our observation that possible SDS-induced changes of the surface protonation state at pH 6 were small enough not to influence the adsorption of EDTA. Considering Equation 1, the observation of constant EDTA adsorption at variable SDS concentrations suggests that SDS should have no effect on EDTA promoted dissolution rates. However, SDS may affect the formation of inner-sphere surface complexes, and hence, the surface speciation of sorbed EDTA, which may potentially affect dissolution rates (vide supra).

While adsorbed SDS had little influence on EDTA adsorption, a strong effect on DFOB and DFOD adsorption was observed (Figure 2). The adsorption of DFOB and DFOD increased strongly with increasing SDS concentrations. Despite their difference in molecular charge, the effects of the anionic surfactant on the adsorption of DFOB^+ ^and DFOD^0 ^were similar.

Cocozza et al. [16] found that DFOB and DFOD form mononuclear inner-sphere surface complexes with a single hydroxamate group coordinating with a surface site. The adsorption of DFOD involves no net change of surface charge in analogy to the adsorption of the monohydroxamate ligand acetohydroxamic acid [17]. The adsorption of DFOB involves the increase of the surface charge due to the positive amine group, probably located relatively far from the goethite surface due to repulsive forces. The difference in charge of these two surface complexes should result in differences in the electrostatic interactions, which is consistent with the observation of somewhat higher adsorption densities of DFOD in the absence of surfactants under otherwise identical conditions [34].

The structures of the siderophores (Figure 1) include a pendant alkyl chain with five carbons giving rise to a local hydrophobic character of the molecule. Bonilha et al. [42] observed that the exchange constant between Na^+ ^and alkylammonium ions (R-NH_3_^+^, with R = C_n_H_2n+1_) in SDS micelles increases with increasing chain size, indicating that hydrophobic interactions contribute to the enhanced exchange constants. Therefore, increased siderophore adsorption in the presence of co-adsorbed SDS suggests that hydrophobic interactions between ligands and surfactants are responsible for the observed behavior. The observed effect of SDS on the adsorption of the two siderophores was similar despite their difference in molecular charge. These observations imply that co-adsorbed surfactants have little influence on electrostatic free energy contributions to siderophore adsorption. This is consistent with our interpretation of the absence of an effect of surfactants on EDTA adsorption as discussed above.

### Adsorption of Fe(III)-complexes

**Figure 3:**
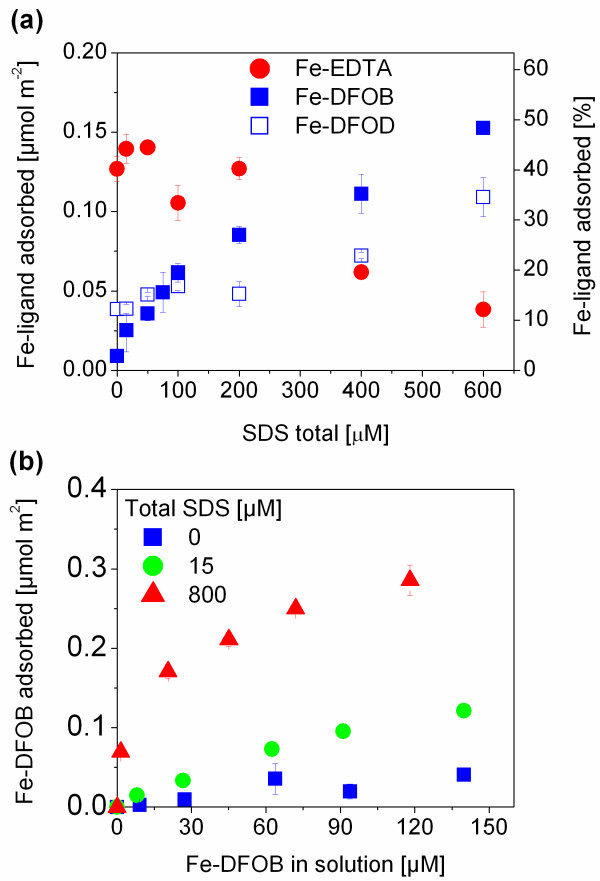
**(a) Adsorption of Fe-EDTA, Fe-DFOB, and Fe-DFOD complexes on goethite at pH 6 as a function of SDS concentration.** The total concentration of Fe-ligand complexes was 30 µM. (b) Adsorption isotherm of Fe-DFOB to goethite at pH 6 in the presence of 0, 15, 800 µM total SDS. For (a) and (b) 0.01 M NaClO_4_, 2.5 g/L goethite, 24 h equilibration time. Error bars indicate the standard deviation of three replicates.

Adsorption of 1:1 Fe(III)-complexes was quantified by measuring the loss of Fe-complexes from solution by UV spectrophotometry and the loss of total Fe by ICP-OES. Both methods gave identical results (see additional file 2), confirming the adsorption of 1:1 Fe-ligand complex. Adsorption equilibrium of Fe-DFOB on goethite was reached after 24 h (see additional file 2). SDS had strong effects on the adsorption of the complexes of DFOB, DFOD, and EDTA (Figure 3a).

The adsorption of the Fe-EDTA complex decreased by almost 75% at the highest SDS concentration relative to adsorption in the absence of SDS. The adsorption behavior of free ligands compared to their iron complexes in the presence of surfactants can be interpreted in terms of their different adsorption modes. In solution, the dominant Fe-EDTA complex is a quinquedentate seven-coordinated bisaquo Fe(III)EDTA^-^·(H_2_O)_2 _with a free carboxylic group [43,44]. Nowack and Sigg [38] suggested that the dominant surface complex at pH values below 7 is an outer-sphere complex (≡FeOH_2_^+^·LFe^-^), while an inner-sphere complex was postulated to dominate at higher pH values (≡FeOFeL^2-^). Therefore, the contrasting effect of SDS on the adsorption of EDTA and its iron complex at pH 6 can be interpreted in terms of their adsorption as inner-sphere and outer-sphere complex, respectively. While the adsorption of the Fe-EDTA complex decreased with increasing SDS concentration (Figure 3a), co-adsorbed surfactants had little effect on free EDTA adsorption (Figure 2). As discussed above, the charge of surfactant aggregates on the goethite surface appeared to have little effect on electrostatic contributions to EDTA adsorption. On the contrary, the Fe-EDTA outer-sphere complex is adsorbed mainly by electrostatic interactions and its plane of adsorption can be expected at greater distance from the mineral surface. Therefore, decreasing adsorption of the negatively charged Fe-EDTA outer-sphere complex with increasing SDS adsorption suggests that electrostatic repulsion between admicelles and the iron complex decrease its adsorption. Nevertheless, some adsorption still occurred at SDS concentrations where electrophoretic mobility measurements indicated charge reversal [21]. This behavior is consistent with the formation of inner-sphere surface complexes (Equation 4) as a minor surface species [38].

In contrast, the adsorption of Fe-DFOB and Fe-DFOD increased over the same range of surfactant concentrations (Figure 3a). The adsorption of Fe-DFOD was higher than that of Fe-DFOB at low surfactant concentrations. At high SDS concentrations, higher adsorption of Fe-DFOB than that of Fe-DFOD was observed. Figure 3b shows the influence of low and high SDS concentrations on the adsorption isotherms of Fe-DFOB on goethite at pH 6. Again, increasing surface concentrations of the Fe-DFOB complex were observed with increasing surfactant concentration. Over a wide pH range (pH 2–11), including the experimental pH of this study, the charge of the iron complexes of the siderophores Fe-DFOB^+ ^and Fe-DFOD^0 ^is the same as the charge of free siderophores [45]. The formation of 6 five-membered rings completely satisfies the coordinative requirements of Fe(III) resulting in extremely stable complexes. Therefore, it is unlikely that the chelated iron adsorbs as inner-sphere surface complex and Fe-DFOB^+ ^and Fe-DFOD^0 ^are expected to interact with the surfactant-coated goethite surface by electrostatic or hydrophobic interactions as outer-sphere surface complexes. Increasing adsorption of both complexes with increasing surfactant concentrations again underlines the importance of the hydrophobic interactions between the hydrophobic backbone of siderophores and the surfactant tail. However, the effect of hemimicelle/admicelle formation on Fe-DFOB^+ ^adsorption was stronger than the effect on Fe-DFOD^0 ^adsorption (higher adsorption of Fe-DFOB^+^, Figure 3a). In contrast to the neutral Fe-DFOD^0^, the adsorption of Fe-DFOB^+ ^benefits not only from hydrophobic interactions but also from electrostatic interactions with the negatively charged admicelle. The positively charged Fe-DFOB^+ ^experiences electrostatic repulsion resulting in low surface concentrations compared to Fe-DFOD^0 ^at small surfactant concentrations. At higher surfactant concentrations (< 100 μM total SDS), Fe-DFOB^+ ^experiences attractive forces due to charge reversal (see electrophoretic mobility in [21]) caused by the formation of hemimicelle and admicelle and its adsorption exceeded Fe-DFOD^0 ^adsorption. This electrostatic effect is not seen in the adsorption of free siderophores forming mainly inner sphere complexes resulting in the location of the charge of the surface complex closer to the mineral surface as discussed above.

Table 1 provides the adsorbed concentrations of SDS and DFOB to goethite at pH 6 as influenced by total SDS concentration in suspension. The ratio Δ(DFOB_ads_)/ΔSDS_ads_) indicates how much additional DFOB was adsorbed with additional adsorption of SDS. These values are only rough estimates, because they are based on SDS adsorption data determined in the absence of the siderophore [21], which was found to have a minor effect on SDS adsorption in preliminary experiments. The strong decrease of the ratio Δ(DFOB_ads_)/Δ(SDS_ads_) with increasing SDS concentration suggests that the effect of adsorbed SDS on the adsorption of DFOB was largest at low SDS surface coverage. Similar differences in ratios were observed for the co-adsorption of fluorocarbon alcohols [46] and 1-pentanol [47] in the presence of adsorbed surfactants on mineral surfaces. These alcohols possess hydrophobic and charged structural moieties that contribute to electrostatic and hydrophobic interactions with adsorbed surfactants, in analogy to the mechanism of siderophore adsorption discussed above. Lai et al. [46] and Lee et al. [47] suggested that low amounts of adsorbed surfactant create two different regions to which amphiphilic compounds can co-adsorb. One region is located between the head groups of the surfactants, and the second region is the hydrophobic perimeter arising from patchwise adsorption of bilayered surfactant aggregates mainly present at low surfactant concentrations [47]. They suggested that the fraction of alcohol adsorbed at the perimeter can be large only at low surfactant concentrations when the aggregates are small and sparse on the mineral surface. Similarly, Asvapathanagul et al. [48], suggested that the structure, nature, or arrangement of adsorbed surfactants at low concentrations had a much stronger effect on co-adsorption of solutes than the actual adsorbed amount. The high Δ(DFOB_ads_)/Δ(SDS_ads_) ratios observed at low SDS loadings suggest that DFOB adsorbs in the two different regions mentioned above.

**Table 1:** Adsorbed amounts of SDS and DFOB on goethite (SDS_ads_, DFOB_ads_) as influenced by the total SDS concentration (SDS_tot_) in suspension at pH 6.

SDS_tot_	SDS_ads_	Δ(SDS_ads_)	DFOB_ads_	Δ(DFOB_ads_)	Δ(DFOB_ads_)/Δ(SDS_ads_)
(μM)	--------------------------- (μmol/m^2^) -----------------------------				
0	0.000	---	0.047	---	---
50	0.054	0.054	0.077	0.030	0.56
100	0.107	0.054	0.091	0.014	0.25
200	0.214	0.107	0.105	0.014	0.13
400	0.439	0.224	0.142	0.037	0.17
600	0.781	0.342	0.171	0.029	0.08

The goethite concentration was 7 g/L and the total DFOB concentration was 80 μM.

The SDS adsorption data were taken from [21] and were measured in the absence of DFOB. Δ(SDS_ads_) and Δ(DFOB_ads_) denote the additional SDS and DFOB adsorbed relative to the next lower SDS_tot _concentration. The ratio Δ(DFOB_ads_)/Δ(SDS_ads_) is an estimate for the additional amount of DFOB adsorbed due to additional adsorption of SDS to the goethite surface.

## Conclusion

Our results demonstrate that adsorbed surfactants can have a strong influence on the adsorption of siderophores and other ligands, as well as the corresponding iron complexes. The degree by which ligand adsorption is impacted by co-adsorbed surfactants depends on their charge, hydrophobicity, and on whether they are adsorbed as outer-sphere or inner-sphere surface complexes. Adsorption of ligands and their Fe-complexes by hemimicelles through hydrophobic and/or electrostatic interactions with the surfactants (here considered as a special case of outer-sphere adsorption) can dramatically change the surface speciation. The altered adsorption of ligands in the presence of surfactants can affect surface controlled processes such as ligand-promoted iron oxide dissolution and the bioavailability of iron. This effect is possibly of high importance not only in soil systems, but also in marine systems where amphiphilic siderophores have been observed [3]. Therefore, the effect of adsorbed surfactants on surface controlled processes can not be neglected in natural environments, where ligands and bio-surfactants are often produced and released together by microorganisms and plants roots.

## Competing interests

The authors declare that they have no competing interests.

## Authors' contributions

NC conducted the adsorption experiments and wrote the first version of the manuscript as part of her PhD thesis, JX synthesized and characterized the DFOD, and SK and RK initiated and supervised the study, and were involved in planning experiments, interpreting data, and revising the manuscript for publication. All authors have read and approved the final manuscript.

## Supplementary Material

Additional file 1 Additional details about the characterization of DFOB by NMR spectroscopy. Click here for file

Additional file 2 Figure showing the adsorption kinetics of Fe-DFOB on goethite. Click here for file
