# Supplementary material for: Adsorption of hydroxamate siderophores and EDTA on goethite in the presence of the surfactant sodium dodecyl sulfate
Source: Geochem Trans. 2009 Jun 13;10:5. doi: 10.1186/1467-4866-10-5 (PMC2706842; doi:10.1186/1467-4866-10-5)
Supplement: Additional file 1 — Additional details about the characterization of DFOB by NMR spectroscopy. [file 1467-4866-10-5-S1.pdf]

Additional File 1

Adsorption of hydroxamate siderophores and EDTA on goethite in the presence of the surfactant sodium dodecyl sulfate

Naraya Carrasco[1], Ruben Kretzschmar*[1], Jide Xu[2] and Stephan M. Kraemer[1,3]

Address: [1]Institute of Biogeochemistry and Pollutant Dynamics, Department of Environmental Sciences, ETH Zurich, CHN, CH-8092 Zurich, Switzerland, [2]Chemistry Department, University of California, Berkeley, CA 94720-1460, USA, and [3]Department of Environmental Geosciences, University of Vienna, Althanstrasse 14, A-1090 Vienna, Austria

* Corresponding author

Characterization of N-acetyl-O,O,O-triacetyl-desferrioxamine and N-Acetyl-desferrioxamine by NMR Spectroscopy

The following section provides some additional details about the characterization of the DFOD by NMR spectroscopy. ^{1}H and ^{13}C NMR spectra were obtained using a Bruker AM-300 NMR spectrometer. NMR chemical shifts are reported in ppm relative to solvent resonance. About 3 mg of sample were dissolved in dueterated dimethylsulfoxide (DMSO-d6) for ^{1}H NMR measurement. For ^{13}C NMR measurements, about 30 mg sample were used each time.

The mass spectroscopy and NMR spectroscopic characterization for N-Acetyl-O,O,O-triacetyl-desferoxamine**:**

MS(FAB+): 729.4 (MH^+).

^{1}H NMR (300 MHz, DMSO-d_6): δ 1.32 (qint, 6H, J = 6.6 Hz, CH_2), 1.48 (mt, 6H, CH_2), 1.53 (m, 6H, CH_2), 1.95 (s, 3H, CH_3), 1.98 (s, 3H, CH_3), 2.19 (s, 9H, CH_3), 2.47 (m, 4H, CH_2), 2.57 (m, 4H, CH_2), 3.18 (qint, 6H, J = 6.0 Hz, CH_2), 3.67 (m, 6H, CH_2), 6.26 (s,br, H, CONH), 6.35 (s,br, H, CONH), 6.43 (s,br, H, CONH).

^{13}C NMR (75 MHz, DMSO-d_6): δ 17.9, 19.8, 22.6, 23.1, 23.3, 25.9, 27.7, 28.5, 29.8, 38.7, 47.0, 168.2, 170.0, 170.3, 171.3, 172.2.

The mass spectroscopy and NMR spectroscopic characterization for N-Acetyl-desferoxamine**:**

MS(FAB+): 603.4 (MH^+).

^{1}H NMR (300 MHz, DMSO-d_6): δ 1.20 (qint, 6H, J = 6.9 Hz, CH_2), 1.36 (qint, 6H, J = 7.0 Hz, CH_2), 1.48 (qint, 6H, J = 7.0 Hz, CH_2), 1.76 (s, 3H, CH_3), 1.95 (s, 3H, CH_3), 2.25 (t, 4H, J = 7.0 Hz, CH_2), 2.56 (t, 4H, J = 7.0 Hz, CH_2), 2.98 (m, 6H, CH_2), 3.44 (t, 6H, J = 6.9, CH_2), 7.78 (m, 3H, CONH), 9.60 (s, 2H, NOH), 9.65 (s, 1H, NOH).

^{13}C NMR (75 MHz, DMSO-d_6): δ 20.4, 22.6, 23.5, 26.0, 27.6, 28.9, 29.9, 38.5, 46.8, 47.1, 169.1, 170.2, 171.4, 172.0.
