# Supplementary material for: Adsorption of hydroxamate siderophores and EDTA on goethite in the presence of the surfactant sodium dodecyl sulfate
Source: Geochem Trans. 2009 Jun 13;10:5. doi: 10.1186/1467-4866-10-5 (PMC2706842; doi:10.1186/1467-4866-10-5)
Supplement: Additional file 2 — Figure showing the adsorption kinetics of Fe-DFOB on goethite. [file 1467-4866-10-5-S2.pdf]

Additional File 2

Adsorption of hydroxamate siderophores and EDTA on goethite in the presence of the surfactant sodium dodecyl sulfate

Naraya Carrasco[1], Ruben Kretzschmar*[1], Jide Xu[2] and Stephan M. Kraemer[1,3]

Address: [1]Institute of Biogeochemistry and Pollutant Dynamics, Department of Environmental Sciences, ETH Zurich, CHN, CH-8092 Zurich, Switzerland, [2]Chemistry Department, University of California, Berkeley, CA 94720-1460, USA, and [3]Department of Environmental Geosciences, University of Vienna, Althanstrasse 14, A-1090 Vienna, Austria

* Corresponding author

Adsorption kinetics of Fe-DFOB on goethite

The required equilibration time for adsorption experiments with SDS and Fe-DFOB was studied in a preliminary adsorption kinetics experiment. Batches of 35.4 mL goethite suspension (2.5 g/L) in 0.01 M $NaClO_4$ were equilibrated at pH 6.0±0.05 with different SDS concentrations for about 68 hours on an end-over-end shaker. Then, 4.6 mL of a Fe-DFOB stock solution (691.5 µM) were added to reach a final Fe-DFOB concentration of 80 µM. The pH was measured and readjusted, if necessary, to 6.0±0.05 by small additions of NaOH or HCl. The final total SDS concentrations were 0, 15, 75, and 600 µM. Over the next 24 hours, 5 mL aliquots of the suspensions were sampled at different time intervals and immediately filtered through 0.025 µm cellulose nitrate membranes. Dissolved iron concentrations were analyzed by inductively coupled plasma optical emission spectrometry (ICP-OES, Vista-MPX, CCD Simultaneous, Varian, Australia).

The adsorption kinetics of the Fe-DFOB complex was investigated in the presence of low and high total SDS concentrations (Figure S2a). Equilibrium was reached faster at low surfactant concentrations. In the presence of 15 or 75 µM total SDS, constant adsorption was reached within 2 hours, while at least 24 h were required to approach steady state in the presence of 600 µM total SDS. Based on these observations, an equilibration time of 24 hours was used for all adsorption measurements. The results showed increasing adsorption of the Fe-DFOB complex with increasing SDS concentration.

The adsorption of the 1:1 Fe(III)-ligand complexes was studied by measuring the total dissolved iron by ICP-OES and the dissolved Fe-ligand complex by UV-visible spectrophotometry during its adsorption on goethite. For both methods the adsorbed concentrations were calculated by mass balance. Figure S2b shows the adsorbed amounts of Fe-DFOB as influenced by increasing SDS concentrations obtained from both analytical methods. Since the adsorption of Fe-DFOB measured as total iron and as iron complex are similar, we assume that Fe in solution is present only as Fe-DFOB complex. Similar results were observed for Fe-EDTA and Fe-DFOD adsorption experiments (results not shown). Based on these experiments, we analyzed Fe-complexes only by ICP-OES in all further adsorption experiments.

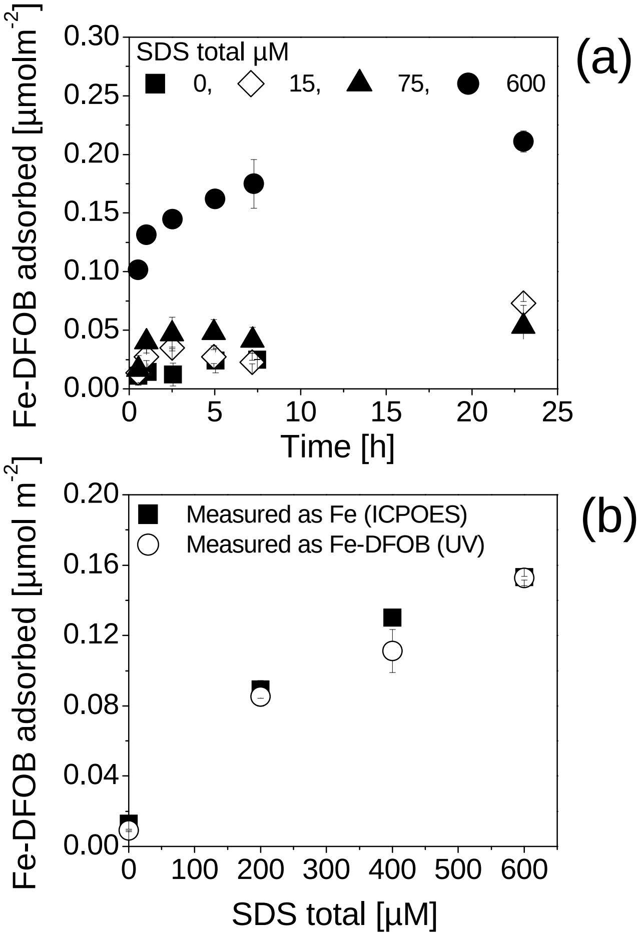

Figure S2

(a) Adsorption kinetics of Fe-DFOB to goethite at pH 6 for different SDS concentrations (80 µM total Fe-DFOB, 0.01 M $NaClO_4$, 2.5 g/L goethite). (b) Adsorption of Fe-DFOB complexes as a function of SDS concentration (30 µM total Fe-DFOB, 0.01 M $NaClO_4$, 2.5 g/L goethite, 24h equilibration time after the addition of Fe-DFOB). Adsorbed amounts were calculated by mass balance based on total iron concentrations measured by ICP-OES or concentrations of Fe-DFOB complexes measured by UV-vis spectrometry. Error bars indicate the standard deviation of three replicates.
